# Performance and simulation analysis of 802.11ax OFDMA in contention-driven scenarios

**DOI:** 10.7717/peerj-cs.2687

**Published:** 2025-02-24

**Authors:** Memoona, Sung Won Kim

**Affiliations:** 1 Information and Communication Engineering, Yeungnam University, Gyeongsan, Gyeongbuk, Republic of South Korea; 2 School of Computer Science and Engineering, Yeungnam University, Gyeongsan, Gyeongbuk, Republic of South Korea

**Keywords:** 802.11ax, OFDMA, ns3, Contention, MU-MIMO, Validation

## Abstract

The 802.11ax standard introduces Orthogonal Frequency Division Multiple Access (OFDMA), shifting the role of access points (APs) in Wi-Fi networks. This shift integrates intricate scheduling logic, assigning coordinator roles to APs for multi-user uplink (MU-UL) transmissions and streamlining downlink traffic flows. These developments require robust network analysis and simulation tools to investigate the trade-offs associated with using OFDMA. In this study, we validate the implementation of OFDMA in ns-3 Wi-Fi module, enhancing flexibility and support for future updates through a redesign process. Previous studies validate the OFDMA implementation in the ns-3 Wi-Fi module by matching the simulation to predictions of analytical models. In this work, we demonstrate that OFDMA performance aligns with analytical predictions through simulation-based performance evaluations using ns-3 in some contention-driven use cases. The proposed system operates in both the uplink (UL) and downlink (DL) directions, implementing two scheduling logics to manage DL traffic flows and coordinate MU-UL transmissions. Simulation time is reduced by introducing parallel computing in the system. This study provides a reliable network analysis and simulation framework that thoroughly examines the trade-offs involved in using OFDMA.

## Introduction

The Wi-Fi Alliance updates the IEEE 802.11ax standard to enhance the network efficiency of 802.11 deployments with devices competing for channel access in various environments, including urban and suburban neighborhoods (Khorov et al., 2019). The introduction of 802.11ax not only improves network efficiency in densely populated environments but also introduces mechanisms to mitigate contention among devices (Magrin et al., 2023). Previous updates to 802.11 primarily focus on boosting peak data rates. In contrast, 802.11ax, commonly known as Wi-Fi 6, prioritizes improving overall network throughput to increase the general speed and effectiveness of wireless communication. Additionally, it promotes more equitable resource sharing within the network, ensuring that services are distributed fairly through improved device coordination. Although 802.11ax outlines several methods to foster device collaboration, details on its implementation are often omitted. The introduction of OFDMA in 802.11ax signifies a shift in the role of the AP in Wi-Fi networks, providing the opportunity to implement more complex scheduling logic to manage DL traffic flows while also acting as a coordinator for MU-UL transmissions (Magrin et al., 2021). Network simulation is a standard technique used to draft and evaluate new ideas, especially when elaborating on details not specified in the specifications. The numerous elements outlined in the standard present challenges for thorough mathematical modeling. While Wi-Fi 6 (IEEE 802.11ax) is relatively new, it has been commercially available since its release in 2019, with products now widely deployed worldwide. As such, creating practical testbeds remains a complex task due to the evolving nature of the technology.

Therefore, simulation serves as a useful bridge, enabling rapid prototyping and affordable testing. To increase confidence in the results and ensure their real-world accuracy, simulation tools must be validated. In this study, we validate the implementation of 802.11ax OFDMA in the ns-3 simulator by comparing simulation results obtained from ns-3 with those predicted by an analytical model in contention-driven scenarios. The proposed analytical model, based on Bianchi’s model, is an enhanced version of other models presented in previous studies (Bianchi, 2000; Bellalta & Kosek-Szott, 2019). We provide a validation method for incorporating 802.11ax OFDMA into the ns-3 simulator. Recent changes, along with an emphasis on increasing feature segregation, lead to a redesign of the Wi-Fi module’s medium access control (MAC) layer architecture. The network performance depends on the scheduler that assigns OFDMA resources to stations (STAs) (Wang & Psounis, 2020). Therefore, we demonstrate that two schedulers, $fbw$ and $fhol$, might achieve different throughput and delay performance.

The rest of this article is organized as follows: “Literature Review” provides the literature review, “802.11ax Advancements” describes recent advancements in 802.11ax, and “MU-MIMO Bandwidth Allocation”, “Efficient Acknowledgment Strategies”, “Dynamic Scheduling in MU DL Transmissions”, “Experimental Configuration”, “Proposed Methodology”, “Validation”, “Conclusions” cover bandwidth allocation, acknowledgment strategies, dynamic scheduling, experimental configuration, proposed methodology, validation, and conclusion.

The contributions of this study are as follows.

- The implementation of OFDMA in the ns-3 simulator validates the IEEE 802.11ax standard.
- The advanced OFDMA implementation in ns-3 improves compatibility and flexibility for future updates. This redesigned and validated implementation provides more accurate and reliable simulation results than previous studies.
- This study validates the performance of OFDMA in contention-driven scenarios, demonstrating its alignment with analytical model predictions—a key novelty of this research.
- The model demonstrates dual-direction operational capabilities. OFDMA is validated in contention-driven scenarios with both UL and DL operations, highlighting its comprehensive capabilities.
- Simulation time, or runtime, is reduced through the incorporation of parallel computing techniques.
- The trade-offs associated with OFDMA usage are comprehensively examined. Consequently, the study provides new insights into the benefits and limitations of the approach under various network conditions.
- This study calculates the efficiency of the OFDMA performance model in single-user (SU) transmission scenarios. This analysis, often overlooked in existing research, is crucial for assessing OFDMA’s real-world applicability and optimization in Wi-Fi networks.

Unlike existing studies, which primarily focus on simplified scenarios for evaluating OFDMA performance, our research introduces and thoroughly analyzes contention-driven scenarios. We conduct a comparative analysis between our contention-driven model and simulation and those from recent studies. Readers are encouraged to refer to the validation section for detailed experimental results and comparisons with previous work.

## Literature review

In business settings where connectivity requirements are high, the effectiveness and dependability of contemporary networks are greatly influenced by wireless communication protocols. This literature analysis combines recent research efforts on evaluating IEEE 802.11 standards with advances in simulation models for performance assessment.

The literature on IEEE 802.11 protocols highlights critical advancements across various network performance aspects, covering topics from deployment issues to optimization techniques in dense network scenarios. Research into IEEE 802.11ac for enterprise networks shows that multiple APs operating on the same channel often lead to low throughput and network unreliability. However, implementing frame aggregation techniques leads to notable throughput improvements (Jonsson et al., 2016). Recent studies emphasize the need to accurately simulate interfering signals in response to receiver capabilities. This approach is essential for high-density vehicular networks, as shown in previous simulations where interference significantly impacts performance (Fuxjäger & Rührup, 2015). Extensive research on OFDMA spectrum sharing reveals that realistic simulations involving a larger number of nodes improve validation of the design (Anchora et al., 2011).

Further advancements in IEEE 802.11ad include performance gains from beamforming and session transfer techniques, with improved reproducibility through public access to model code (Assasa & Widmer, 2016). Comparative studies show that IEEE 802.11ac outperforms 802.11n in speed, latency, and jitter due to advanced features such as guard intervals and channel bonding (Ravindranath et al., 2016). In the context of the Internet of Things (IoT), IEEE 802.11ah leverages sub-1-GHz communications to address network challenges effectively, and simulations capture realistic protocol behavior (Tian et al., 2016). IEEE 802.11ax introduces OFDMA, which facilitates optimal resource unit allocation even in dense environments. This approach enhances random access efficiency by optimizing the number of resource units, as shown in one study (Uwai et al., 2016). Another study on OFDMA technology for high-density WLANs in IEEE 802.11ax demonstrates that it improves connection density, reduces access delays, and increases throughput (Brahmi, Yazid & Omar, 2020).

Scheduler optimization in IEEE 802.11ax is crucial for uplink (UL) transmissions, with proposed schedulers significantly boosting data flow speeds (Bankov et al., 2018). Additionally, the combination of OFDMA and MU-MIMO in IEEE 802.11ax enhances both downlink (DL) and uplink (UL) throughput, with notable efficiency gains in UL transmission, despite challenges in collision rates (Daldoul, Meddour & Ksentini, 2020). For real-time applications, IEEE 802.11ax’s uplink OFDMA, combined with a resource allocation algorithm (CRA), enables ultra-low latency and high reliability, showing promising simulation results (Avdotin et al., 2019).

Innovative schemes such as MUSE leverage uplink OFDMA and MU-MIMO to enhance multi-user transmission efficiency, collecting channel state information (CSI) to optimize downlink MU-MIMO groupings, thereby maximizing system utility (Lee, 2019). Research into advanced scheduling strategies underscores the importance of CSI utilization for resource unit allocation, particularly under 802.11ax constraints (Wang & Psounis, 2020). Finally, simulations of mixed traffic in dense environments, including overlapping basic service sets (OBSS), explore the impact of DL-UL traffic on overall network performance (Kim, 2017). These studies enhance our understanding of wireless network standards and modeling approaches. They highlight the importance of accurate simulation modeling for practical evaluation and provide useful information about the performance characteristics of IEEE 802.11 standards across diverse network scenarios.

## 802.11ax Advancements

Wi-Fi 6, commonly referred to as 802.11ax, disrupts the field of wireless communication. Its primary objective is to boost per-user throughput and the overall effectiveness of local area networks.

By introducing novel features, 802.11ax enhances network efficiency and spatial reuse through optimized channel access coordination. The goal of this technology is to completely transform how devices manage channel access accomplished by introducing two revolutionary features: OFDMA and BSS color.

**Figure 1:**
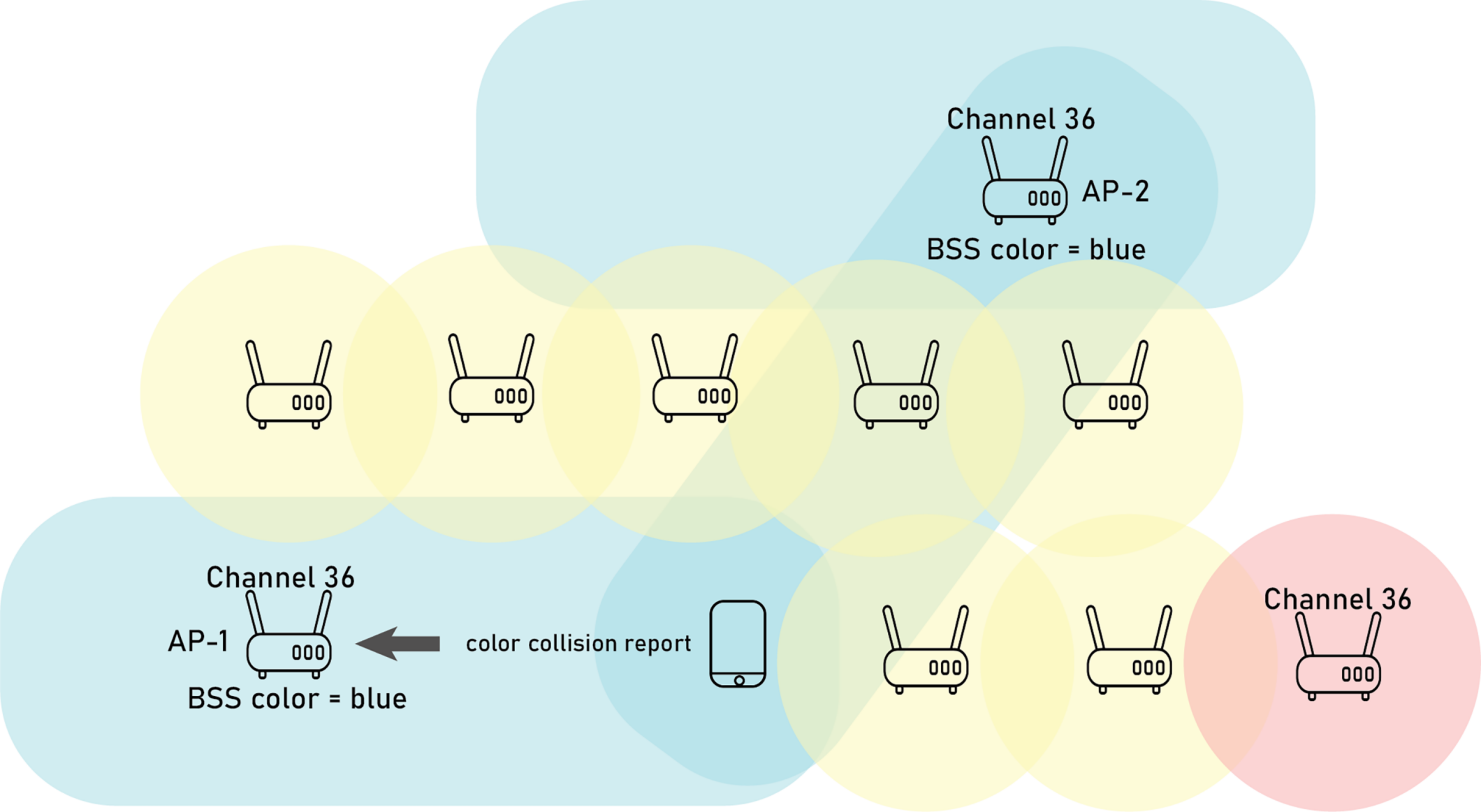
Example of BSS coloring collision.

The BSS coloring mechanism in IEEE 802.11ax is designed to improve spatial reuse in dense network environments by mitigating interference between overlapping BSSs (OBSSs). Each BSS is assigned a unique “color,” which essentially tags its frames with an identifier to distinguish it from nearby networks. This allows devices to make more informed decisions about accessing the medium, especially in high-density scenarios. BSS color also reduces interference between OBSSs. In situations where older Carrier-Sense Multiple Access with Collision Avoidance (CSMA/CA) access protocols consider the channel busy, BSS color allows devices to proceed with data transmission within the same BSS. It empowers APs to effectively coordinate transmissions to and from multiple STAs within the same BSS, particularly in OBSSs. In 802.11ax, an AP can adjust its BSS color if it detects a color collision with an overlapping BSS (OBSS) using the same color. This detection may come directly from the host AP or through reports from client stations. A client’s autonomous report contains information about all detectable OBSS BSS colors. For instance, as illustrated in Fig. 1, AP-1 may not detect the color of AP-2, but a client associated with AP-1 could detect the OBSS with the same color and send a color collision report.

The AP can then broadcast this color change to all connected clients through a BSS color change announcement in an Action frame. This updated color information may also be shared *via* beacons, probe responses, or reassociation response frames, ensuring that all associated devices are informed of the new BSS color setting.

This strategic cooperation enhances the network’s spatial reuse and spectral efficiency. The advent of OFDMA marks a paradigm shift from a distributed strategy to a hybrid medium access model, allowing APs to act as network managers through enhanced distributed channel access (EDCA). APs now optimize DL and UL transmissions by allocating OFDMA subcarriers to specific STAs in blocks called RUs.

**Figure 2:**
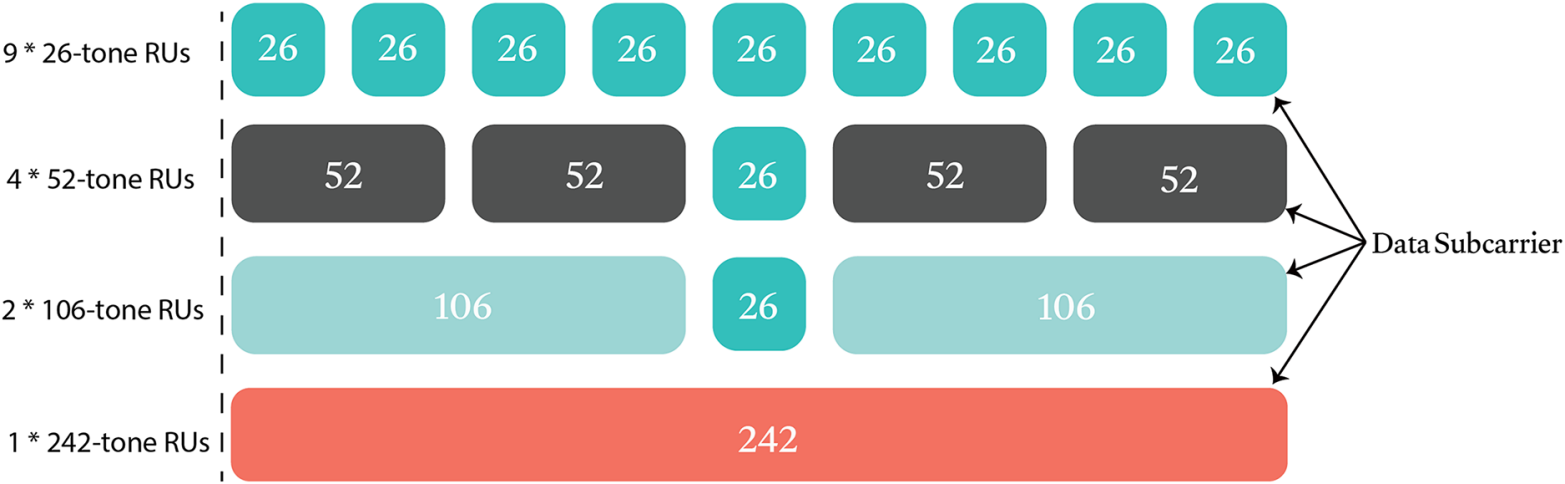
Some RU allocations in a 20 MHz channel.

**Figure 3:**
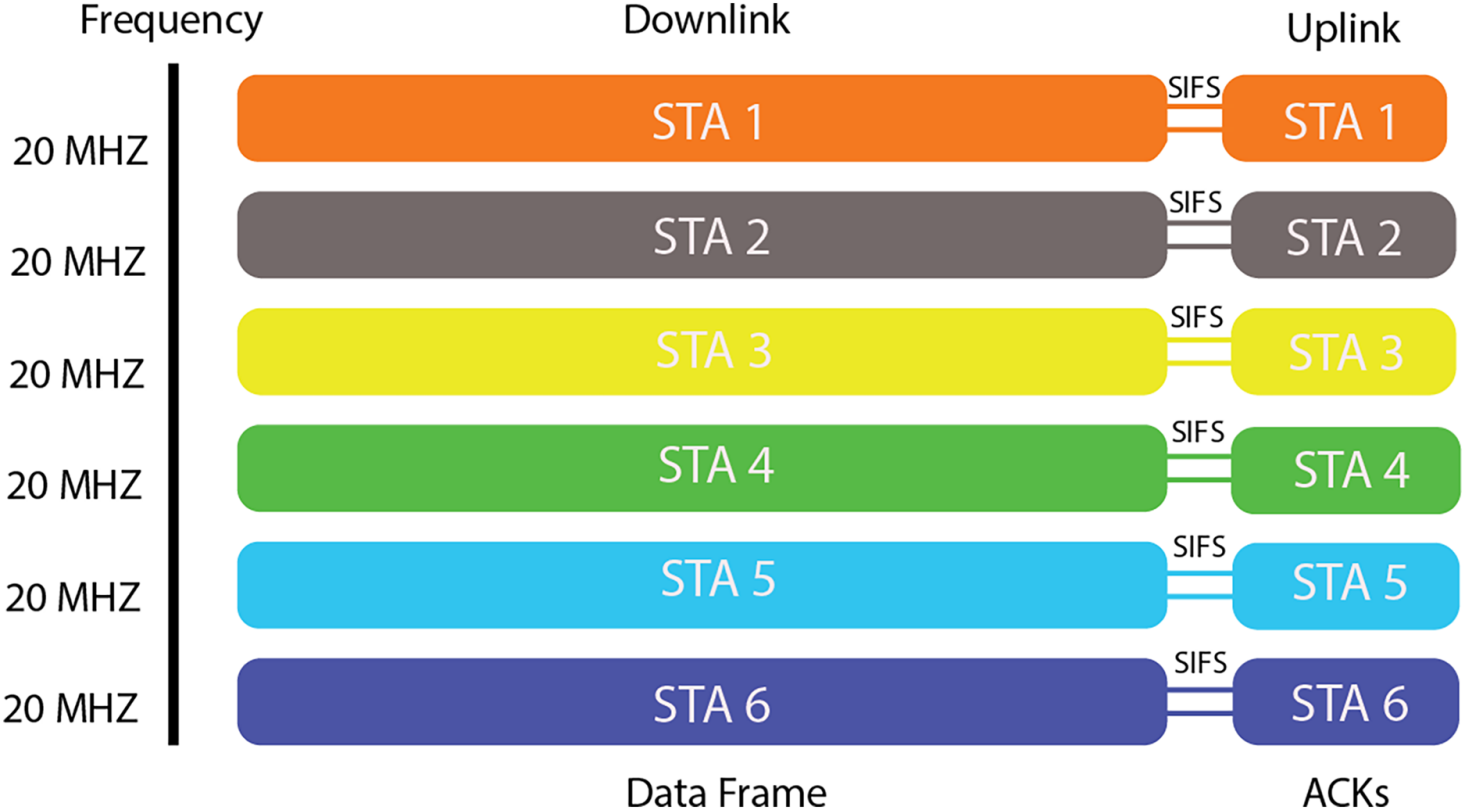
OFDMA transmission.

Figure 2 illustrates the possible RU allocations in a 20-MHz channel. During a single transmission opportunity (TXOP) after channel access, APs efficiently serve numerous STAs, eliminating the need for recurring contention and simplifying the transmission process. Figure 3 illustrates the downlink and uplink transmission process for multiple stations (STAs) over a 20 MHz channel in an IEEE 802.11ax network, showcasing the role of Short Interframe Space (SIFS) intervals between the data frame and acknowledgment (ACK) responses.

Multiple-input and multiple-output (MIMO) technology, initially introduced by 802.11ac, is further enhanced in 802.11ax to boost downlink multi-user (MU) transmission. This advancement improves both throughput and scalability for DL MU-MIMO, which is known for optimizing frequency utilization efficiency (Yamada, Denno & Hou, 2023). This eliminates the need for separate CSI collection for MU broadcasts and complements spatial separation for distinct STAs in the frequency domain by separating transmissions. The High-Efficiency (HE) WLAN standard 802.11ax ushers in a new era in wireless communication, prioritizing network efficiency through sophisticated spatial reuse mechanisms.

OFDMA and BSS Color allow APs to maximize channel access and tackle interference issues, resulting in overall performance improvement. Wi-Fi 6 is widely adopted for its unmatched efficiency, increased throughput, and diverse connectivity paradigm, offering a superior wireless experience.

## Mu-mimo bandwidth allocation

While serving MU OFDMA, the AP carefully distributes the available bandwidth, as discussed in Naik, Bhattarai & Park (2018). For simplicity, this study assumes that the bandwidth B can only be divided into RUs of equal size, which represents a refinement of the foundational work proposed by Karaca et al. (2016). The function $f(B,N)$, where *N* represents the number of STAs, determines the distribution of total bandwidth *B* among several STAs. This function outputs the number of RUs into which the whole bandwidth *B* is divided.

For example, if $N = 1$ and $B = 20$ MHz, during a transmission opportunity (TXOP), only one STA has access to the entire 20 MHz channel, promoting a more distributed usage of the available bandwidth. In a different scenario during TXOP, if $f$ is set to 1 and *B* is adjusted to 10 MHz, a single STA has complete access to the whole 10 MHz channel. When *N* increases, a vital adjustment in the allocation occurs, potentially causing some STAs to lose service, thereby demonstrating the optimization of resource utilization.

To facilitate the negotiation of a trade-off between spectral efficiency and device compatibility, a higher $f$-value is necessary. For example, $f = 5$ indicates a coarser bandwidth partition with fewer RUs but serves more STAs. The scheduling logic determining which STAs to service is independent of the exact definition of $f$. This study assumes that STA services are supplied in a round-robin fashion to facilitate a cyclical and sequential process of STA priority. This systematic scheduling strategy ensures that resources are distributed equitably among the STAs, preventing any single STA from monopolizing resources in the long-term. This methodology is a crucial part of the overall network optimization strategy and promotes equitable allocation. Two straightforward schedulers are considered for analysis and simulation.

Let $fbw$ be the scheduling function that maintains the equal-sized RU requirement and avoids RU wastage by finding the minimum number of RUs required to serve the maximum number of STAs.

For example, if $B = 20$ MHz and $N = 5$ STAs, then $fbw = 3$ ensures that each station has an RU of approximately 6.7 MHz. Additionally, RUs with a size of 5.2 MHz are used to ensure that four STAs are supplied concurrently for $N = 8$:

$$fbw(20,8) = 4.$$

Another scheduling function, referred to as $fhol$, is designed to accommodate the maximum number of STAs within the given TXOP. For example, if $N = 10$, then:

$$fhol(20,10) = 5.$$

This shows that each station receives an RU with a size of 4 MHz for each MU DL transmission. Table 1 displays the results with both scheduling functions. Finally, it is critical to emphasize that designing better schedulers becomes more practical when we move away from the assumption that all RUs must have the same size.

**Table 1:** Scheduling functions.

N	1	2	3	4	5	6	7	8	9
$fbw$	1	2	2	4	5	5	5	5	9
$fhol$	1	2	4	6	9	9	9	9	9

This decision facilitates the cooperation of analytical and simulation methods in both cases: when the AP utilizes the available bandwidth and when it decides to spare some RUs. This decision simplifies collaboration in both cases.

## Efficient acknowledgment strategies

Acknowledgments (ACKs) are effectively managed using a complex technique in response to MU-UL broadcasts, which are crucial for preserving communication integrity. The AP ensures that all linked STAs receive ACKs simultaneously in DL OFDMA. This technique increases the overall efficiency of the communication network and optimizes the acknowledgment process, as demonstrated in the works of Kosek-Szott & Domino (2022) and Daldoul, Meddour & Ksentini (2020). However, the intricate nature of ACKs creates challenges after MU DL broadcasting. After the DL broadcast, a sequence of STAs is synchronized, and each STA must send the ACK back to the AP. This ACK collection mechanism must be coordinated to ensure the stability and reliability of the communication system (Naik, Bhattarai & Park, 2018). This research thoroughly examines three distinct ACK sequences to better understand and analyze the underlying dynamics. These sequences are meticulously recreated in both analytical and simulation models.

### ACK-SU-format

Following the MU DL transmission, this protocol adds more layers of complexity and optimization to the acknowledgment process, making it a sophisticated cornerstone in the complex environment of this study. The AP methodically initiates an event chain within a precisely timed framework. After the transmission, it sends a well-crafted SU block ACK request (BAR) to every STA, starting a series of alternating UL and DL transmissions. These transmissions occur within the TXOP of the AP, following a sequential deployment process. When each STA returns its unique SU ACK to the AP, the intricate nature of this synchronization is revealed. The emerging sequence of successive transmissions results in a coherent convergence of acknowledgments, precisely placed within the TXOP. The intricacy of the protocol, which is thoroughly validated and implemented in both simulation and analytical models, reflects its utility and the scope and depth of this study (Bellalta & Kosek-Szott, 2019).

### MU-bar

This protocol is essential for optimizing MU-DL transmission. After transmitting the MU BAR to the device group participating in the MU DL transmission, the AP systematically establishes a short inter-frame space (SIFS).

One of the distinctive features of the MU BAR is its traffic frame, which is designed to accurately schedule and manage the simultaneous transmission operations of all associated STAs. This innovative approach improves the performance and reliability of the wireless network, ensuring robust communication and reducing the likelihood of contention issues. The internal processes of the protocol are carefully assessed while integrating it into analytical and simulation models to confirm the efficacy of the MU-BAR procedure in practical situations (Bellalta & Kosek-Szott, 2019). Furthermore, with different quality of service (QoS) requirements, the concept of MU-BAR can be expanded to explore its potential applications.

### AGGR-MU-BAR

This protocol aggregates the MU BAR proactively and directly at the end of the MU DL transmission by the AP. This integration facilitates the relaying of ACKs by STAs during a SIFS, simplifying the communication procedure immediately after the transmission. This efficient and synchronized coordination reduces delays and increases the wireless network’s speed and reliability.

The complexity of the aggregate MU-BAR (AGGR-MU-BAR) protocol is systematically included in analytical and simulation models, extensively validated, and demonstrated to work in practical circumstances (Bellalta & Kosek-Szott, 2019).

## Dynamic scheduling in mu dl transmissions

The ability of an AP to carry out MU DL broadcasts in the dynamic sphere of wireless network operations demonstrates the sophistication attained in contemporary network management. By utilizing the capabilities of HE MU physical-layer (PHY) protocol data units (PPDUs), this process goes beyond simple data transfer and explores the realms of strategic network resource optimization and user-centric service delivery.

The true complexity lies in the vendor-specific design of scheduling algorithms. The foundational standards for MU transmissions provide a structure for exchanging messages. These operational tools are designed to manage the comprehensive interactions between user behavior, wireless channel conditions, and network requirements. The role of the AP in this scenario is complex. It involves the logical distribution of RUs according to the requirements of each STA, careful selection of subsets of STAs for service, and meticulous calibration of the MCS for every transmission. The AP’s capacity to collect and evaluate real-time data, including the complex signal-to-noise ratio (SNR) of received packets, traffic requirements, and access categories (AC) of the network traffic, further enhances this decision-making process. The AP requests and analyzes channel quality data from the STAs, adding an additional layer of intelligence through feedback mechanisms. This enables the AP to optimize its MCS selections for optimal communication efficacy.

The advanced HE trigger-based (TB) PPDU structure facilitates MU-UL coordination process through the AP’s strategic decision-making. This period of network functioning is characterized by the synergy of simultaneous UL broadcasts from several STAs, all expertly arranged by the deployment of a trigger frame (TF). The Special Frame contains essential scheduling information that ensures organized and efficient communication. This scheduling information includes the following: duration of the UL HE TB PPDU, RU allocation, and transmission power. The TF, which is the central component of this intricate coordination, contains a multitude of scheduling information, including the careful placement of RUs and the length of the UL HE TB PPDUs.

When a TF is received, the participating STAs initiate their synchronized UL transmissions following an SIFS while adhering to the scheduling guidelines specified in the TF. The selection of an MCS for these UL transmissions is equally important and requires a trade-off between throughput goals and channel conditions. The strategic depth observed in the DL OFDMA scenario mirrors the AP’s role in creating a TF, with the added complexity of imperfect real-time knowledge about each STA’s queue. Buffer status report polls (BSRPs) are commonly used to allow the AP to request and process queue status data from the STAs, leading to more informed and efficient scheduling decisions. RUs are allocated as access slots under the control of the AP. If a STA does not have a specific RU (Gan, 2015), it chooses a slot arbitrarily, with the transmission carried out using a planned operation known as the OFDMA back-off (OBO) protocol (Yang, Deng & Chen, 2017). When a STA cannot make the most of the allotted transmission time, padding techniques are used. Resources must be allocated carefully to consider the design of UL OFDMA transmissions, which allocate a defined time for each participating STA. This ensures that network resources are preserved and the transmission schedule is executed automatically.

## Experimental configuration

The ns-3 802.11 simulator differs from the MATLAB WLAN toolbox by focusing on the simulation of complex networks with numerous devices competing for access. Instead of detailed physical layer simulations, it employs simplified models to represent the performance of the PHY layer (Henderson et al., 2008). Ns-3 is a well-maintained and extensively used simulation tool in research, valued for its flexible architecture, detailed documentation, and open-source nature (Kumar et al., 2015).

### Simulation study description

Throughout this study, the ns-3 simulation script models an AP serving multiple 802.11ax STAs, also called HE STAs. The script’s complex structure and abundance of features enable control over network activities.

The acceptance and rejection of the use of OFDMA in the DL and UL directions are the major functions of the AP and critical components of the configuration. When the MU DL functionality is enabled, the AP always transmits DL packets using OFDMA. This strategic deployment enables multiple STAs to receive DL packets simultaneously whenever possible. If not, SU transmissions are utilized. In the framework of testing, user datagram protocol (UDP) traffic is generated at a predefined set rate by the AP and STAs, with one acting as the destination for the other. A formal block ACK agreement is carefully established between the AP and each STA at the beginning of the simulation. This agreement dictates the subsequent ACK sequence employed. To promote consistency in the wireless communication environment, the AP and STAs within the system share the same transmission power levels. Detailed simulation parameters are provided in Table 2.

**Table 2:** Simulation parameters.

Parameter	Value
Network radius	15 m
AP antenna gain	5 dbi
STA antenna gain	3 dbi
MCS	5
APP layer traffic rate	100 Mbit/s
Traffic type	UDP
Channel bandwidth	20 MHZ
Frame aggregation size	40,960 ms
MSDU lifetime	25,000 ms
Simulation time	8 s
Propagation loss model	Two-ray ground

### The scenario description

In the revalidation scenario, the key metrics are head-of-line (HoL) delay and throughput. The analysis considers various factors, including the number of STAs, the type of scheduler employed, channel access parameters, the ACK sequence, and the intensity of both DL and UL traffic. Furthermore, to assess the robustness of OFDMA and its practical applications, this study investigates the impact of contention on these metrics.

### The schedulers description

In IEEE 802.11ax, OFDMA provides subchannel allocation by the APs, referred to as resource units (RUs), to serve multiple STAs in parallel. Scheduling decisions regarding these RUs utilize various algorithms to optimize performance metrics such as throughput and delay while managing contention for the shared medium. In this regard, we implement two straightforward schedulers, referred to as fbw and fhol, which offer different methodologies for RU allocation. Each of these schedulers balances bandwidth usage and transmission delay differently, yielding varying throughputs and latency performance based on network and traffic conditions. The fbw scheduler aims to maximize bandwidth efficiency by optimizing the use of available RUs. This translates to serving as many STAs as possible within a given TXOP with minimal wastage of RUs, thereby reducing how many times the AP contends for the channel and optimizing channel utilization over time. On the other hand, the fhol scheduler is designed to minimize delay for STAs. It seeks to reduce the waiting time between successive transmissions to the same STA, allowing each STA to gain more frequent access in the available TXOPs. As a result, this scheduler sacrifices some bandwidth efficiency to achieve lower average delays. For their detailed functionality, please refer to “MU-MIMO bandwidth allocation”.

### The analytical model description

The analytical model is based on the Bianchi IEEE 802.11 Distributed Coordination Function (DCF) model, which was developed for random access in legacy Wi-Fi, that is, non-OFDMA. To accurately capture the specifics of OFDMA-based transmissions, including multi-user (MU) capabilities and AP-initiated MU transmissions, the model expands to incorporate the unique features of IEEE 802.11ax. This involves calculating all types and sizes of OFDMA-specific transmissions, such as MU DL and UL transmissions, and adding new parameters introduced by OFDMA overhead, like channel sounding. The proposed extension to Bianchi’s model enables the study of OFDMA behavior in IEEE 802.11ax. The model is designed for a scenario in which a single access point (AP) serves N stations exclusively using the best effort (BE) access category (AC). This means queues remain consistently full in both the UL and DL directions, and all transmitted packets share the same priority. Thus, all nodes, including the AP and STAs, continuously maintain packets in their buffers for transmission. The model incorporates key variables such as slot transmission probability, OFDMA and legacy OFDM symbol durations, channel bandwidth, contention levels, number of back-off (BO) stages, collision probability, and the likelihood of successful transmission during empty slots.

## Proposed methodology

The proposed methodology in this study evaluates a communication system’s overall performance metrics using a rigorous computational technique. This study combines theoretical modeling with real-world applications to understand the complexities of system efficiency and behavior.

### Problem formulation and core function definition

First, the problem is formulated to understand how a communication system performs under different operational settings. To this end, a core function called $core \; function(x)$ is defined. This function captures the system’s behavior and serves as the foundation of the computational analysis.

### Fixed point iteration method

The fixed point iteration function from the scipy.optimize library is used to iteratively solve for the system’s parameters. This iterative process identifies the optimal parameter values that reflect the system’s behavior, ensuring convergence to precise solutions. The algorithm incrementally adjusts parameters, reducing error margins at each step. The robustness of this method allows it to handle non-linearities within the system, ensuring that the final parameter values are both reliable and accurate.

### Computation of transmission and collision probabilities

The proposed system calculates collision and transmission probabilities across various transmission scenarios in the communication system, considering aggregation levels, channel access probabilities, and transmission speeds.

### Formulation of relevant formulas and equations

Equations are formulated to accurately simulate the system’s behavior. These mathematical expressions include those for the probability of successful transmissions

$$P_{{\mathrm{coll}}}^{{\mathrm{STA}}} = 1 - \left( {1 - {\gamma _{{\mathrm{AP}}}}} \right) \cdot {\left( {1 - {\gamma _{{\mathrm{STA}}}}} \right)^{{M_{{\mathrm{STA}}}} - 1}}$$

where ${\gamma _{{\mathrm{AP}}}}$ and ${\gamma _{{\mathrm{STA}}}}$ represent the transmission probabilities for the AP and STA, respectively, and ${M_{{\mathrm{STA}}}}$ denotes the count of STAs in the network, including transmission timings

$${T_{{\mathrm{SU - TX}}}} = {T_{{\mathrm{SU - Data}}}} + {T_{{\mathrm{IFS}}}} + {T_{{\mathrm{SU - ACK}}}}$$

where ${T_{{\mathrm{SU - Data}}}}$ denotes the duration for data transmission, ${T_{{\mathrm{IFS}}}}$ represents the inter-frame spacing time, and ${T_{{\mathrm{SU - ACK}}}}$ is the duration of the acknowledgment message, followed by throughput estimations.

$${\mathrm{Throughpu}}{{\mathrm{t}}_{{\mathrm{DL}}}} = {{{b_1}{k_{{\mathrm{SU}}}}C + {b_3}{Z_u}{k_{{\mathrm{MU - DL}}}}C} \over \psi }$$

where *C* is the size of the payload, ${k_{{\mathrm{SU}}}}$ and ${k_{{\mathrm{MU - DL}}}}$ denote the number of frames in SU and MU DL transmissions, ${Z_u}$ represents the number of destination STAs, and $\psi$ is a factor based on event probabilities and timing, and collision probabilities for both UL and DL broadcasts

$${P_{{\mathrm{coll,SU}}}} = \alpha \cdot {\gamma _{{\mathrm{AP}}}} \cdot {P_{{\mathrm{fail,AP}}}},\quad {P_{{\mathrm{coll,MU}}}} = (1 - \alpha )\beta \cdot {\gamma _{{\mathrm{AP}}}} \cdot {P_{{\mathrm{fail,AP}}}}$$

where ${P_{{\mathrm{coll,SU}}}}$ and ${P_{{\mathrm{coll,MU}}}}$ represent the probabilities of collision for SU and MU downlink transmissions, and ${P_{{\mathrm{fail,AP}}}}$ is the collision probability for the AP.

### Assessment of system parameters and metrics

The study provides a comprehensive analysis of the influence of different system features on performance metrics such as channel utilization, throughput, and latency. The research systematically modifies aggregation levels, transmission rates, and device types to observe their effects on system efficiency.

### Computation of head-of-line delays

HOL delay computation is an integral part of this study as it enables the assessment of the system’s queuing behavior. In other words, it helps evaluate the system’s ability to handle concurrent transmissions, manage packet traffic, and effectively reduce contention.

### Validation and comprehensive analysis

All relevant indications and aspects are thoroughly examined to validate the computational results. This examination includes comparing the computational outputs to theoretical expectations and testing them against current empirical data. This validation method ensures that the results are consistent and accurate.

### Interpretation and conclusion

This article concludes by explaining the significance of communication systems design and optimization and their computational interpretations. The analytic findings are combined to establish conclusions about system performance, efficiency, and potential areas for improvement.

### Algorithm description

Algorithm 1 computes the transmission probabilities, contention probabilities, and throughput for UL and DL links in a network. It employs a core function and a fixed-point iteration mechanism while determining the HOL delay using the parameters provided. The core function calculates the transmission and contention probabilities for APs and HE STAs. The core function is defined, and the number of HE STAs is initialized (lines 2–3 in the algorithm). The function E calculates the expected contention using the contention window, maximum backoff (BO) stage, and contention probability in lines 4–5. Line 6 calculates the contention probability for the AP. Contention probabilities for APs and HE STAs are calculated based on the transmission probabilities of the other type of node (lines 7–8). Transmission probabilities for APs and HE STAs are calculated based on the expected contention windows and contention probabilities (lines 10–13). The fixed-point iteration method is used to solve for stable transmission and contention probabilities iteratively until convergence. An initial guess array for the fixed-point iteration is initialized with zeros. The scipy.optimize fixed point function is used to solve the core function for stable probabilities (lines 17–18). Transmission timings for different scenarios are calculated to determine the time required for data transmission. Transmission timings for single- and MU scenarios are estimated using the time required for data transmission, ACK, and BO (lines 21–23). The probabilities of specific outcomes in a defined time slot are computed to understand the likelihood of successful transmission. These probabilities are calculated based on the transmission and contention probabilities, as well as the number of devices and network configuration (lines 26–35). Throughput for both UL and DL is calculated to understand the data transfer rate. Throughput times for different scenarios are computed based on the transmission times and probabilities. The computed throughput is aggregated to calculate the overall throughput. The HOL delay is used to assess the time delay experienced by data packets in the network. The delay is estimated using the transmission scenario and the number of devices in the network (lines 38–50). Finally, the computed metrics and relevant parameters are output for analysis and interpretation. Algorithm 1 shows the core function for computing transmission probabilities and throughput calculation.

**Algorithm 1:** Core function for computing transmission probabilities and throughput calculation.

**Require:** Input parameters *x*
**Ensure:** Computed values for *t*_*ap*_, ${t_{he\_sta}}$, ${p_{coll\_cont\_ap}}$, ${p_{coll\_cont\_he\_sta}}$
1: # Initialize core function for computing transmission probabilities
2: ${t_{ap}},{t_{he\_sta}},{p_{c\_ap}},{p_{coll\_cont\_he\_sta}} \leftarrow x$
3: $nu{m_{he\_sta}} \leftarrow NumHe$
4: **function** E( $Cont{W_{min}},m,{p_c}$)
5: **return** $\displaystyle{{(1 - {p_{coll\_cont}} - {p_{coll\_cont}} \cdot {{(2 \cdot {p_{coll\_cont}})}^m})} \over {(1 - 2 \cdot {p_{coll\_cont}})}}$
6: **end function**
7: $\displaystyle {p_{coll\_cont\_ap}} \leftarrow 1 - {(1 - {t_{he\_sta}})^{nu{m_{he\_sta}}}}$
8: **if** $nu{m_{he\_sta}} \; > \; 0$ **then**
9: ${p_{coll\_cont\_he\_sta}} \leftarrow 1 - (1 - {t_{ap}})$
10: **else**
11: ${p_{coll\_cont\_he\_sta}} \leftarrow 0$
12: **end if**
13: ${t_{ap}} \leftarrow \displaystyle{1 \over {E(Cont{W_{min\_ap}},ma{x_{ap}},{p_{coll\_cont\_ap}}) + 1}}$
14: **if** $nu{m_{he\_sta}} \; > \; 0$ **then**
15: $\displaystyle{t_{he\_sta}} \leftarrow {1 \over {E(Cont{W_{min\_he\_sta}},ma{x_{he\_sta}},{p_{coll\_cont\_he\_sta}}) + 1}}$
16: **else**
17: ${t_{he\_sta}} \leftarrow 0$
18: **end if**
19: $ outcome \leftarrow [{t_{ap}},{t_{he\_sta}},{p_{coll\_cont\_ap}},{p_{coll\_cont\_he\_sta}}]$
20: **return** *outcome*
21: # Fixed Point Iteration
22: $initial\_guess \leftarrow$ array of zeros with size 4
23: $result \leftarrow$ scipy.optimize.fixed_point(core_function, initial_guess)
24: $\displaystyle probability \leftarrow {{(1 - {p_{coll\_cont\_ap}} - {p_{coll\_cont\_ap}} \cdot {{(2 \cdot {p_{coll\_cont\_ap}})}^{ma{x_{ap}}}})} \over {(1 - 2 \cdot {p_{coll\_cont\_ap}})}}$
25: # Transmission times
26: $T{r_{su}} \leftarrow (T{r_{su\_Du}} + T{r_{SIFS}} + T{r_{BACK}} + T{r_{SIFS}})$
27: $T{r_{mu\_du}} \leftarrow get\_T{r_{mu\_du}}(nu{m_{a\_mu\_dl}},{V_u})$
28: $T{r_{mu\_u}} \leftarrow maxTxopDuration$
29: # Probabilities of having a certain outcome at a given slot
30: $nu{m_{he\_sta}} \leftarrow NumHe$
31: $\displaystyle{p_{coll\_he\_device}} \leftarrow {{NumHe} \over {params[^\prime nStations^\prime ]}}$
32: $a1 \leftarrow ({t_{ap}} \cdot {p_{coll\_he\_device}} \cdot \alpha \cdot (1 - {p_{coll\_cont\_ap}}))$
33: $a2 \leftarrow (nu{m_{he\_sta}} \cdot {t_{he\_sta}} \cdot (1 - {p_{coll\_cont\_he\_sta}}))$
34: $a3 \leftarrow ((1 - \alpha ) \cdot \beta \cdot {t_{ap}} \cdot {p_{he\_device}})$
35: $a4 \leftarrow ((1 - \alpha ) \cdot (1 - \beta ) \cdot {t_{ap}} \cdot {p_{he\_device}})$
36: $b1 \leftarrow ((1 - {t_{ap}}) \cdot {(1 - {t_{he\_sta}})^{nu{m_{he\_sta}}}})$
37: ${p_{coll\_he\_tx}} \leftarrow (1 - {(1 - {t_{he\_sta}})^{nu{m_{he\_sta}}}})$
38: # UL and DL throughput
39: $T{h_{a1}} \leftarrow T{h_{su}}$
40: $T{h_{a2}} \leftarrow T{h_{su}}$
41: $T{h_{a3}} \leftarrow T{h_{mu\_d}}$
42: $T{h_{a4}} \leftarrow T{h_{mu\_u}}$
43: $T{h_{c1}} \leftarrow T{h_{cont\_su}}$
44: $T{h_{c2\_sta}} \leftarrow T{h_{cont\_su}}$
45: $T{h_{c2\_ap}} \leftarrow T{h_{cont\_mu\_dl}}$
46: $T{h_{c3\_sta}} \leftarrow T{h_{cont\_su}}$
47: $T{h_{c3\_ap}} \leftarrow T{h_{cont\_mu\_ul\_ap}}$
48: $T{h_{c4}} \leftarrow T{h_{cont\_su}}$
49: # Head-of-line
50: $hol \leftarrow {\bf{if}}\;params[^\prime dl^\prime ] = = \;^\prime\!\!\! su ^\prime \;{\bf{then}}\;NumHe \cdot (T{h_{su}} + {E_{ap}} \cdot T{h_e}) \cdot 1000\;{\bf{else}}\;{{NumHe} \over {Va{r_u}}} \cdot (T{h_{mu\_d}} \;\;+ {E_{ap}} \cdot T{h_e}) \cdot 1000$

## Validation

**Figure 4:**
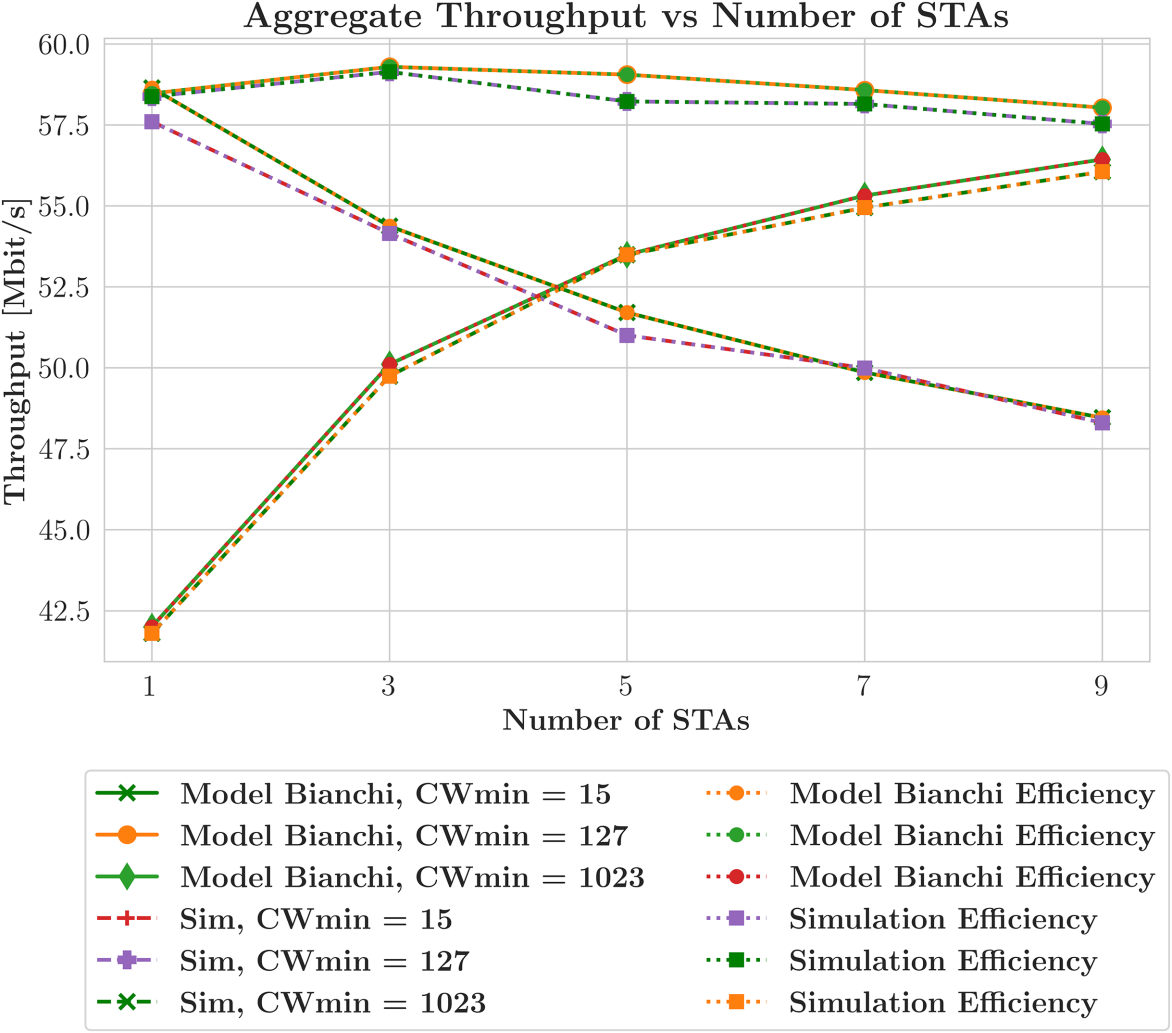
Aggregate throughput efficiency in single user (SU) transmissions (MCS5, 20 MHz).

**Figure 5:**
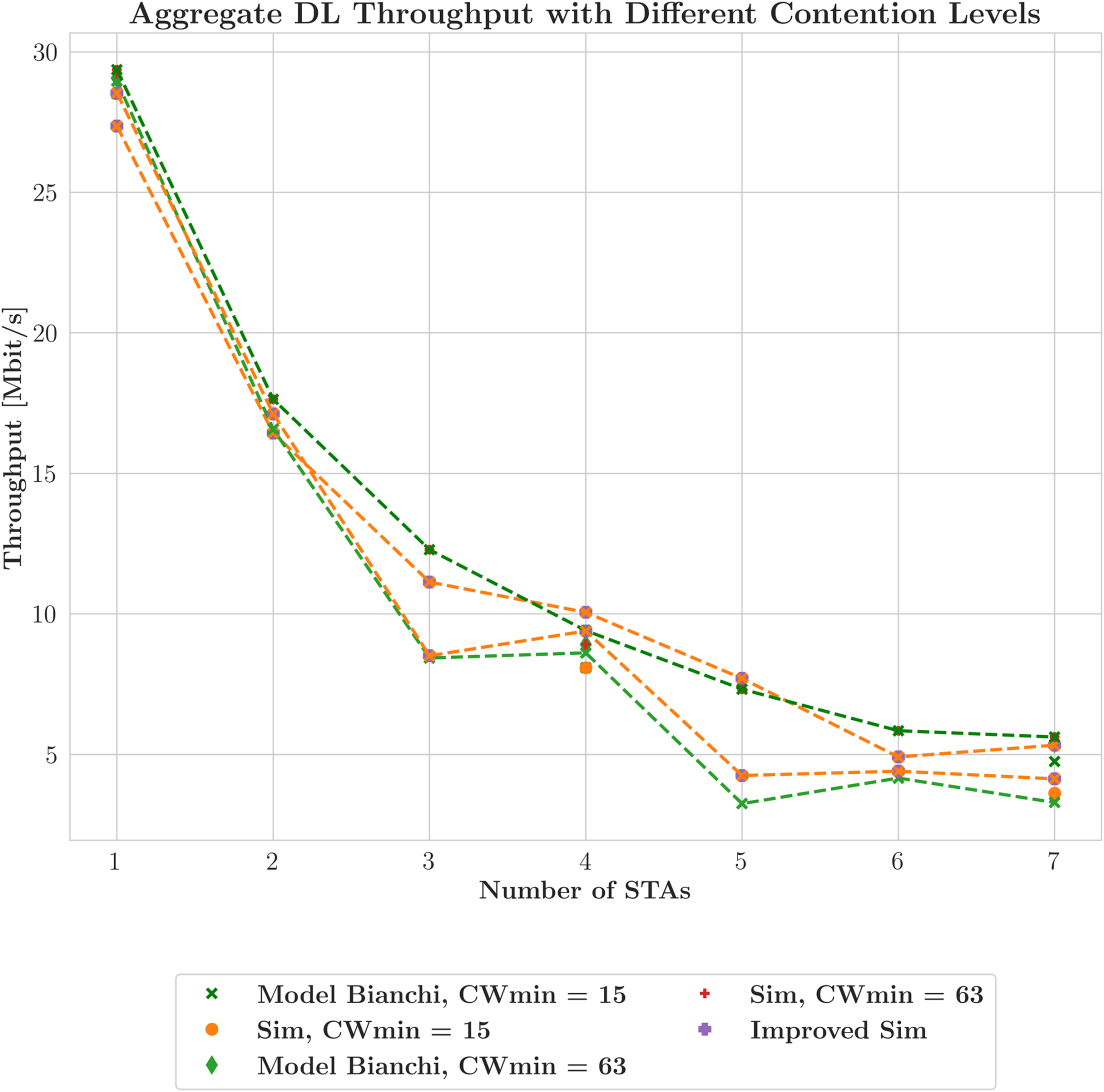
Downlink aggregate throughput at varying contention levels with bidirectional traffic (MCS5, 20 MHz).

To evaluate the performance of our proposed work, we conduct a thorough comparative analysis against a well-established method (Bellalta & Kosek-Szott, 2019). We organize the findings comprehensively in Table 3 to present the results of our comparative analysis. The simulated scenario is described in “Experimental Configuration” and summarized in Table 2. Figure 4 illustrates the aggregate throughput of SU transmissions. It compares the model predictions, represented as solid lines, with the simulation results, represented as dashed lines. The proposed analytical model demonstrates the closest relationship with independently derived computer simulations compared to other analytical models from the literature and computer simulations based on an NS-3 simulator model. In Fig. 4, the X-axis represents the number of STAs, and the title refers to ’throughput with efficiency’ as the figure includes both throughput and efficiency metrics. Each throughput line has a corresponding efficiency line shown as a dotted line in the same color. This pairing illustrates the achieved throughput along with the efficiency as a percentage of the theoretical maximum—the highest possible throughput that can be attained under ideal conditions. This reveals the correlation between the total network throughput and the number of STA terminals. Empirical observations indicate that the model makes accurate predictions, as these lines consistently converge under various contention window settings. In addition to throughput, the network’s efficiency is also assessed, as indicated by the circular and rectangular shapes. Network performance is evaluated using efficiency statistics, considering resource utilization in addition to raw throughput. Resource utilization and increased contention, driven by a higher number of STAs, are the main factors that reduce efficiency. Three different values of the CWmin parameter—15, 127, and 1,023—are analyzed to cover a broad spectrum of potential network contention scenarios. The effect of the contention window on network performance is reflected by the CWmin value associated with each pair of throughput and efficiency curves, demonstrating the closeness of the mathematical model results to those of the simulation and emphasizing the importance of considering both throughput and efficiency when assessing network performance. The study results, described in “Validation”, are depicted in Fig. 5, which demonstrates the model and simulation estimates for DL throughput when two schedulers are utilized, focusing on MU broadcasts. This analysis focuses on a network configuration where all the devices are HE STAs generating traffic and where the devices are configured to utilize the AGGR-MU-BAR ACK sequence with an additional contention window. Contention effects on the ( $fbw$) scheduler are illustrated by greater, more frequent horizontal gaps between the orange lines due to re-transmissions and collisions. These gaps represent the larger intervals between successful transmissions for each STA. While attempting to balance between throughput and fairness, $fbw$ does not allocate minimizing re-transmissions the same priority as $fhol$. Consequently, compared to $fhol$, it only slightly improves contention handling. $fhol$ manages contention marginally better than $fbw$ due to its emphasis on reducing re-transmissions.

**Table 3:** Comparative analysis.

Proposed work	Bellalta & Kosek-Szott (2019)	Key findings
Validates ns-3’s throughput performance under Single User (SU) transmissions, showing scalability as the number of STAs increases in dense environments.	Describes an UL transmission scenario with MU-MIMO and OFDMA but does not provide specific throughput validation for SU setups.	ns-3 validation confirms robust throughput performance, even in high-density SU scenarios.
Validates aggregate downlink (DL) and uplink (UL) throughput for mixed traffic, demonstrating ns-3’s efficiency in managing simultaneous DL and UL traffic.	Provides DL and UL throughput rates for SU *vs*. MU configurations, focusing on AP-initiated transmissions, without mixed traffic validation.	ns-3 shows robust throughput in mixed DL/UL traffic, outperforming AP-initiated approach’s scope.
Analyzes Head-of-Line (HoL) delay under Multi-User (MU) downlink traffic, highlighting ns-3’s capability to minimize latency in high-density settings.	Compares aggregate DL and UL throughput but does not provide a specific latency metric or delay analysis, focusing instead on overall throughput.	ns-3 provides precise latency analysis, essential for real-time applications in dense WLANs.
Examines how different acknowledgment (ACK) sequences affect downlink throughput, demonstrating ns-3’s flexibility in configuring efficient ACK handling.	Analyzes throughput sensitivity to parameters $\alpha$ and $\beta$ but does not focus on acknowledgment sequences or their impact on performance.	ns-3 validation offers adaptable ACK strategies to optimize throughput, outperforming fixed settings.
Shows how varying contention window sizes impact DL throughput, with higher contention leading to reduced throughput.	Investigates the impact of increasing A-MPDU size on DL and UL throughput, showing potential gains from larger aggregations.	Provides practical insights into optimizing throughput in high-density networks with contention.
Examines Head-of-Line (HoL) delay across different contention levels, comparing delay-optimized and throughput-optimized schedulers.	Examines how increasing channel width enhances DL and UL throughput, particularly with MU configurations.	Offers actionable data on delay-optimized scheduling for real-time applications.
Analyzes DL throughput across ACK sequences under various contention windows, showing optimal ACK strategies for high efficiency.	Studies throughput gains from adding more antennas at the AP, improving spatial diversity and overall performance with increased streams.	Demonstrates how optimized ACK sequences can improve throughput in contention-heavy networks.

**Figure 6:**
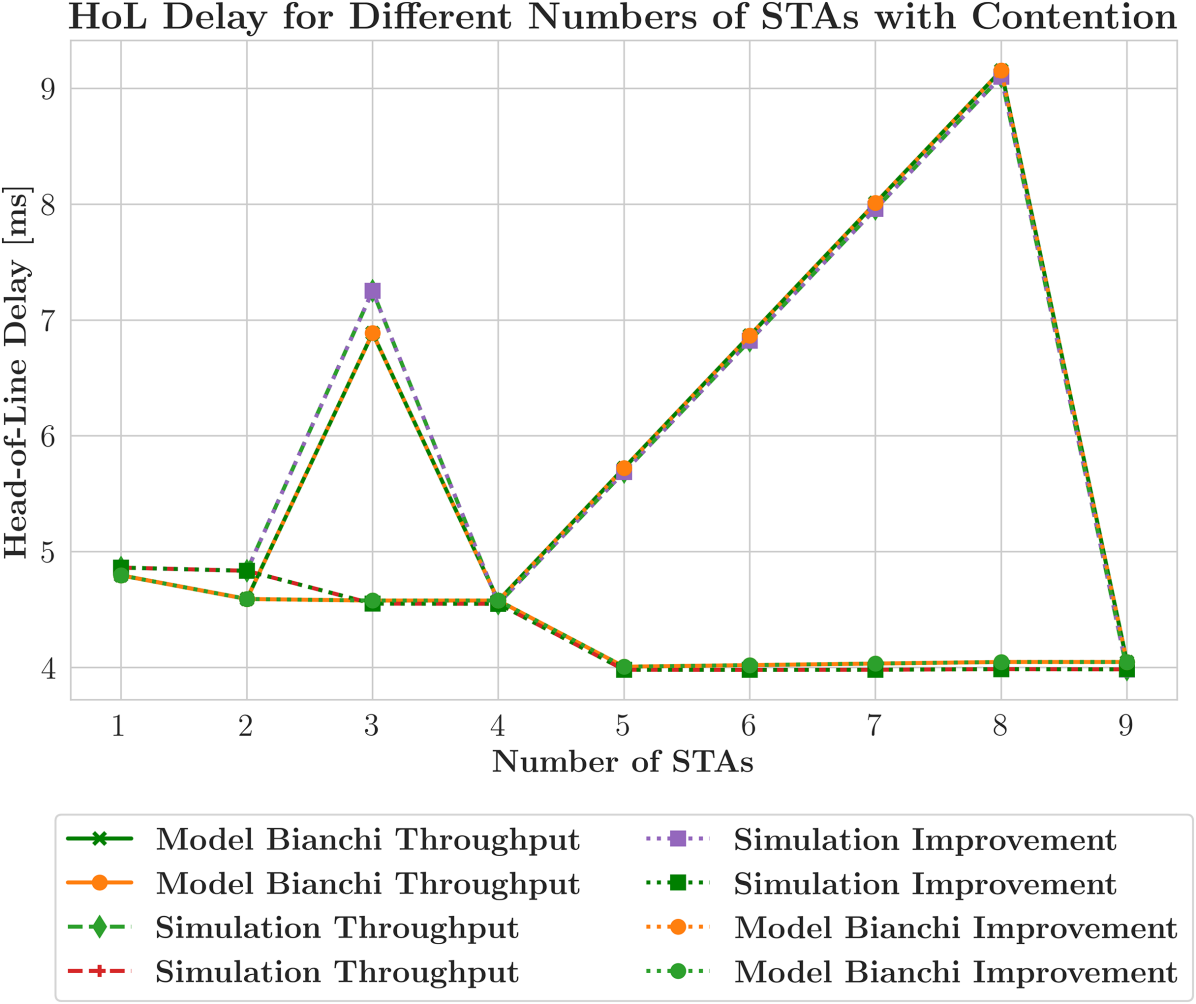
Head-of-line (HoL) delay across station densities with contention (MCS5, 20 MHz).

As observed from the graph’s smaller gaps between the red lines, this is reflected in the more gradual increments in the HoL delay relative to $fbw$. Nonetheless, $fhol$ continues to prioritize reducing delay before increasing throughput. As observed in the graph, the performance differences between the schedulers are most notable for STA values other than 1, 2, 4, and 9. This situation results in more pronounced differences in their throughput responses to contention because the RU allocation functions differ. The plot confirms the overall finding that, under contention, $fhol$ exhibits marginally greater resilience to HoL delay than $fbw$. However, this leads to additional reductions in throughput. Figure 6 illustrates the effect of contention on the HoL delay in an OFDMA network using two distinct scheduling strategies, $fhol$ and $fbw$. The study incorporates a contention window to simulate channel resource contention, based on a previously specified scenario that includes HE STAs, DL traffic, and the AGGR-MU-BAR ACK sequence. The addition of a contention window significantly impacts the performance of both schedulers. The simulation throughput for the fhol scheduler is represented by a red dashed line, whereas the throughput for the fbw scheduler is represented as a green dashed line with diamond markers. The throughput of the Bianchi model that uses the fbw scheduler is illustrated by an orange solid line with circular markers, whereas the throughput for the Bianchi model that uses the fhol scheduler is shown as a green solid line with cross markers. Additionally, model improvements with the fbw scheduler are depicted by an orange dotted line with circular markers, while those with the fhol scheduler are illustrated by a green dotted line with circular markers. Simulation improvements using the fbw scheduler are indicated by a purple dashed line with rectangular markers, and those with the fhol scheduler are marked by green rectangles. In this context, the $fbw$ scheduler results in longer intervals between consecutive retransmissions to the same STA due to increased channel contention, while the $fhol$ scheduler achieves lower average delays between consecutive transmissions to the same STA. Collisions and re-transmissions significantly increase the HoL delay.

**Figure 7:**
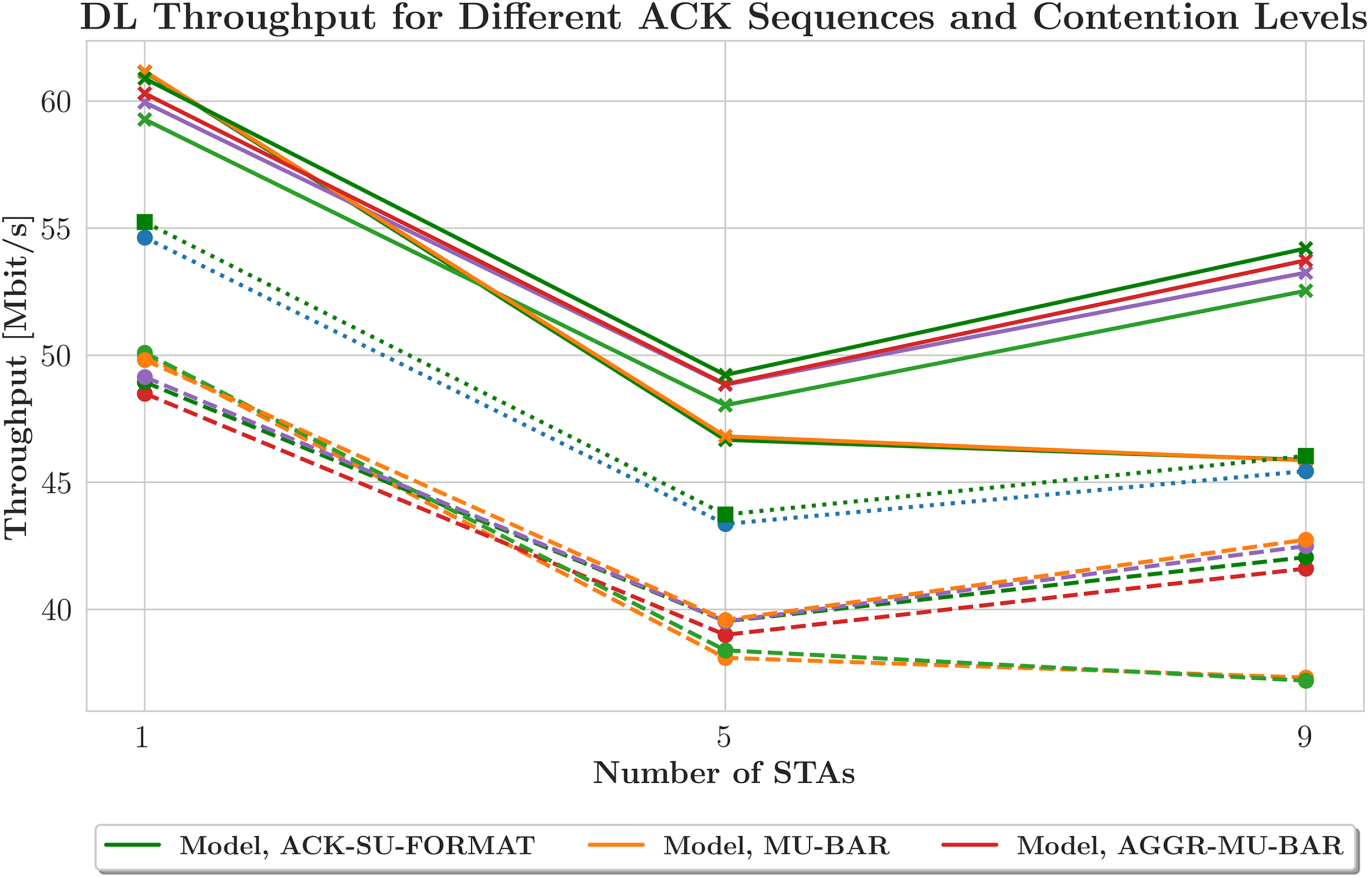
Downlink throughput across acknowledgment (ACK) schemes under contention.

**Figure 8:**
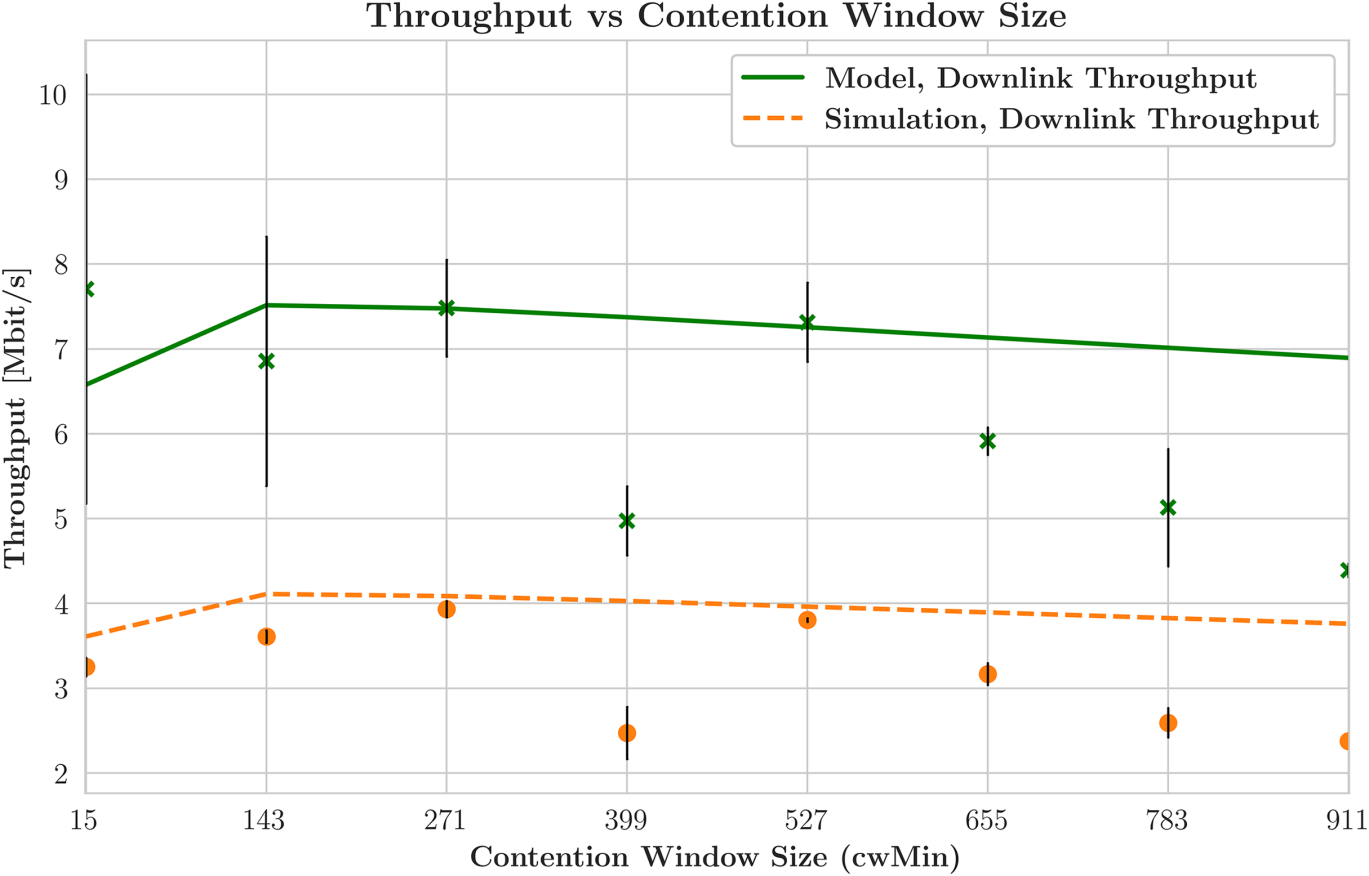
Aggregate downlink throughput across contention values.

While $fbw$ attempts to balance throughput and HoL delay, its limited focus on reducing retransmissions results in only marginally better performance than $fhol$ in terms of contention. In contrast, $fhol$ encounters conflict with more resistance as it prioritizes reducing retransmissions. Notable differences in scheduler performance emerge when the number of STA values falls outside 1, 2, 4, 9, where the RU allocation functions yield divergent outcomes. For instance, with the number of STAs equal to 3 in a contention-based environment, the $fhol$ scheduler allocates resources to serve all three STAs within a single transmission opportunity (TXOP) by dividing the available bandwidth into 4 RUs. This approach ensures that the average HoL delay aligns with the average interval between consecutive TXOPs at the AP. In contrast the $fbw$ scheduler omits one STA from each TXOP, thereby extending the average time between consecutive DL transmissions within this contention scenario. Figure 7 displays the model and simulation assessments of DL throughput under various ACK sequences. The AP serves as the only source of data transmission in these simulations, with each STA receiving traffic at a rate of 1 Mbit/s. The saturation problem that occurs with the use of a small number of STAs in this arrangement, which encounters a contention window causing contention, is prevented. The simulation results (orange line with “x” marker) are compared with the estimates produced by the model (green line with circles). Generally, $fbw$ shows greater throughput than expected and focuses on maximizing throughput, except for a few STA values (1, 2, 4, and 9). Due to increased contention, the total DL throughput drops as the number of STAs rises. The orange line’s “x” marker represents improvements in the simulation outcomes over an earlier run by reducing variability and more closely matching the model’s predictions for the number of STAs. The throughput stabilizes after a brief decline. This pattern suggests that the network adapts to the increased traffic, and STAs likely use effective backoff techniques influenced by the contention window configuration. Additionally, the issue of network saturation is mitigated by efficiently handling the contention parameters. Figure 8 depicts a thorough analysis of this study, achieved by incorporating a contention window into the process, enabling the distinction between schedulers that prioritize delay (shown in orange) and those that prioritize throughput (shown in green). The graph of contention window size *versus* throughput reveals a steady decline in both the simulated and modeled datasets. This pattern highlights that, as the contention window size increases, throughput decreases notably. The annotated crosses and dots on the modeled data points indicate the inherent diversity of the model-predicted throughput values. Detailed findings point to a possible discrepancy between the simulated and actual throughput, which may vary within the range indicated by these variabilities.

**Figure 9:**
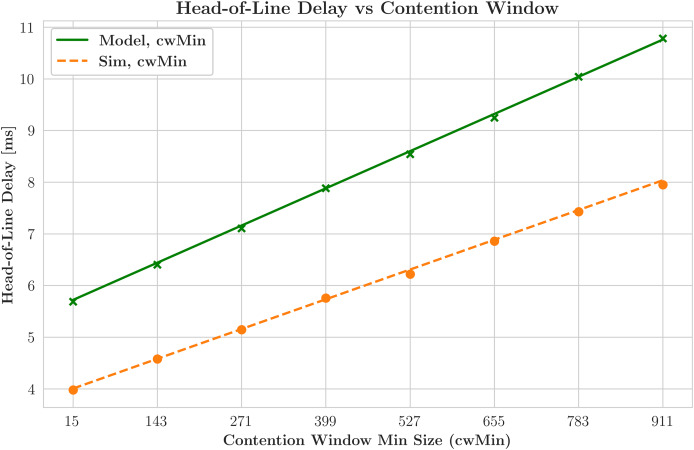
Head-of-line (HoL) delay variation with contention levels.

Figure 9 examines the relationship between the contention window and HOL delay by displaying the smallest contention window sizes, which range from 15 to 911, along the x-axis. A clear pattern is visible in both simulated and model-derived data, indicating that the HOL delay increases as the contention window increases. This outcome is expected, as longer wait times between device transmission attempts during extended contention windows lead to increased delays. The comparative analysis demonstrates that a delay-optimized scheduler outperforms its throughput-optimized counterpart regarding HOL delay. Because delay-centric schedulers are efficient at controlling the transmission time, there is less variation in the number of packets waiting at the front of the transmission queue.

**Figure 10:**
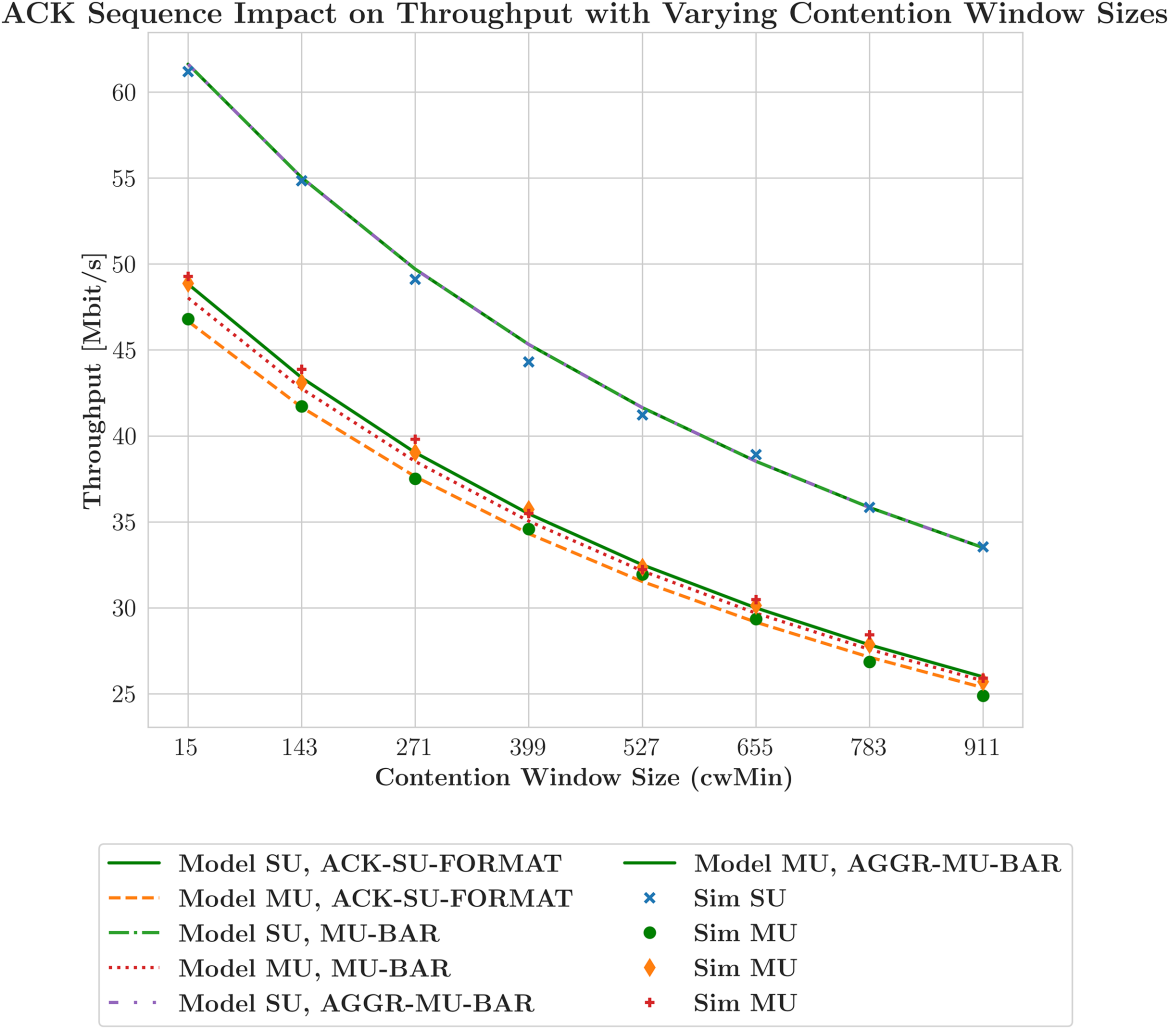
Downlink throughput performance by ACK scheme and contention levels.

Conversely, the scheduler that prioritizes throughput, represented by the green line, exhibits a higher HOL delay because it emphasizes maximizing data transfer rates. This prioritization may lead to longer wait times, particularly in cases where certain STAs are excluded from the transmission schedule. Figure 10 illustrates the relationship between contention window size and throughput achieved with various ACK techniques, featuring several lines that represent different network models and their corresponding simulated data. This graph reveals a significant deviation from earlier observations. Simulations indicate a decrease in throughput with smaller contention window sizes, which occurs because the network operates below saturation thresholds. This situation suggests that the STAs cannot generate sufficient traffic to fully utilize the network’s capabilities, leading to a divergence from the model’s assumption of saturation conditions. The average packet delay increases with the number of STAs for both schedulers due to contention. As more devices compete for the same channel resources, the transmission times for packets lengthen. The $fbw$ scheduler consistently exhibits higher average packet delays than $fhol$. This difference likely occurs because $fbw$ prioritizes throughput more than other protocols and may experience a higher number of retransmissions due to collisions during contention. The general trends followed by the orange and green lines indicate that the model’s estimations accurately reflect the behavior of the analyzed system.

## Conclusions

In this study, we validate the ns-3 implementation of 802.11ax OFDMA by comparing the simulation output with the results from a previously validated model for contention-driven scenarios. We align the throughput and average HoL delay results across a range of parameters. The findings conclude that OFDMA demonstrates superior performance even under rigorous contention scenarios. Future work can further enhance the study by considering adaptive resource allocation techniques that incorporate RUs of different sizes or alternative approaches.

## Additional Information and Declarations

### Data Availability

The following information was supplied regarding data availability:

The code is available at Zenodo: memoonaqayyum. (2024). memoonaqayyum/Performance-and-Simulation-Analysis-in-Contention-Driven-Scenarios: v1.0.0 (v1.0.0). Zenodo. https://doi.org/10.5281/zenodo.14046465.
